# Cognitive task analysis and workload classification

**DOI:** 10.1016/j.mex.2021.101235

**Published:** 2021-01-19

**Authors:** Benjamin M. Knisely, Janell S. Joyner, Monifa Vaughn-Cooke

**Affiliations:** Department of Mechanical Engineering, University of Maryland, College Park, MD 20740, United States

**Keywords:** Human performance, Workstation, Autonomous, Pupillometry, Human-machine system

## Abstract

Automation can be utilized to relieve humans of difficult and repetitive tasks in many domains, presenting the opportunity for safer and more efficient systems. This increase in automation has led to new supervisory roles for human operators where humans monitor feedback from autonomous systems and provide input when necessary. Optimizing these roles requires tools for evaluation of task complexity and resulting operator cognitive workload. Cognitive task analysis is a process for modeling the cognitive actions required of a human during a task. This work presents an enhanced version of this process: Cognitive Task Analysis and Workload Classification (CTAWC). The goal of developing CTAWC was to provide a standardized process to decompose cognitive tasks in enough depth to allow for precise identification of sources of cognitive workload. CTAWC has the following advantages over conventional CTA methodology:

• Integrates standard terminology from existing taxonomies for task classification to describe expected operator cognitive workload during task performance.
• Provides a framework to evaluate adequate cognitive depth when decomposing cognitive tasks.
• Provides a standard model upon which to build an empirical study to evaluate task complexity.

## Graphical abstract

**Figure:**
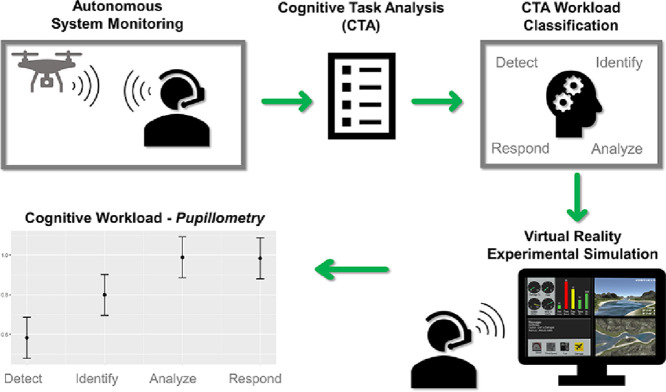

Specifications table

**Table:** 

Subject Area:	Engineering
More specific subject area:	*Human Factors*
Method name:	Cognitive Task Analysis and Workload Classification (CTAWC)
Name and reference of original method:	*Cognitive Task Analysis*[1]
Resource availability:	

## Introduction

Automation is progressively taking over roles once allocated to humans, presenting opportunities to relieve the burden of strenuous and repetitive tasks, and to create more efficient systems. As a result, new supervisory roles are being created where humans actively observe autonomous systems and provide input when needed. These new supervisory roles, where operators are continuously receiving system output and responding accordingly, are highly variable, existing across many domains, and ranging in complexity. This variability in complexity can influence the performance of the supervisory tasks. It is not fully understood how to evaluate and optimally design these new roles to maximize human and system performance.

Cognitive task complexity, due to its unobservable nature, is not particularly well understood. Cognitive tasks of varying complexity can lead to varying degrees of cognitive workload in humans. Cognitive workload is a latent construct that describes the effort required by the working memory to perform a cognitive task [2]. Workload can be measured in many ways, including self-reporting [3,4], performance measures (accuracy and timing of tasks) [5,6], behavioral observations [7], and neurophysiological measures such as pupil response [8,9], heart rate variability [10], EEG [11,12], and core temperature [13].

Cognitive workload can influence human performance in different ways. Excessive cognitive workload has been associated with poor human performance and error [4,^14^,15], while moderate levels of elevated cognitive workload has been linked to increased performance [16,17]. One recent study found that large, sudden spikes in cognitive workload corresponded to decreased performance, whereas consistent, elevated cognitive workload corresponded to increased performance [18]. Regardless, understanding the level of workload that operators experience in complex systems is critical to optimize the system for human performance. A method to classify tasks based on cognitive workload can provide a basis for evaluation. This work provides a methodological approach to accomplish this. Before tasks can be classified, an approach to decompose a high-level system goal into meaningfully distinct sub-tasks is required.

Task analysis is an analytical process in which a skill, movement, or cognitive process is decomposed into sub-tasks that a system operator must complete to accomplish the high-level goals of the system [19]. Task analysis is a useful process because it facilitates the identification of task subcomponents which can be evaluated or modified independently. It is a hallmark tool in human factors research, and has been used in many applications including product design, instructional design and training, function allocation, and error and workload assessment [20]. In this paper, we are primarily interested in performing task analysis on cognitive tasks, known as cognitive task analysis (CTA).

CTA focuses on the underpinning mental framework, thought processes, and knowledge behind the performance of a task [1]. It can be used to identify hidden and ineffective cognitive strategies as well as tasks that induce high cognitive demand. It can also be used as a baseline model for task optimization to maximize human performance [21,22]. Approaches to CTA are very diverse, with researchers having identified over 100 different varieties [1]. Five steps common to most CTA approaches are: 1) Collect preliminary knowledge; 2) Identify knowledge representation; 3) Apply focused knowledge elicitation methods; 4) Analyze and verify acquired data; and 5) Format results for intended application [1]. Approaches are typically classified by the knowledge elicitation approach, and generally includes 1) Observation and interviews; 2) Process training; and 3) Conceptual techniques [23]. CTA has been used in a variety of domains, including autonomous vehicle display design [24], electronic health record design [25], instructional design [26,27], and design of medical training programs [28].

In this paper, a new approach to CTA is discussed that includes several improvements over existing approaches. Existing CTA methodologies do not represent cognitive tasks in adequate cognitive depth and lack standardization. Cognitive depth permits precise identification of sources of cognitive workload. Standardized terminology to describe cognitive tasks can facilitate comparison between analyses and provide a theoretical framework for continued validation. Additionally, few methods have attempted to integrate CTA results into empirical research and use that data to perform task-specific cognitive workload analysis. Chan & Kaufman [29] utilized standard terminology from Bloom's taxonomy to classify tasks, however no empirical validation of the classification was performed. Liang et al. [30] performed CTA and rated the cognitive workload demand of tasks using the VACP rating scale [31] and empirically validated the ratings with data obtained via NASA-TLX. While this work did contain a validation of standardized classifications for task cognitive workload, metrics for validation were limited to subjective survey data collected post hoc. An approach for task cognitive workload validation that integrates objective (accuracy, timing) and neuro-physiological (e.g. pupil dilation) data into the validation process could provide additional robustness. This paper discusses an approach to CTA that uses standardized terminology from existing cognitive and psychomotor taxonomies to describe operator cognitive workload and a process to empirically validate and analyze task-specific cognitive workload.

## Methods

The following section introduces a CTA methodology to predict operator cognitive workload entitled Cognitive Task Analysis and Workload Classification (CTAWC). The steps of this methodology are as follows:

1) Traditional Task Analysis – The task or process of interest is decomposed into basic actions the user performs to achieve the end goal. This is performed iteratively with help from users and expert stakeholders.
2) Cognitive Task Analysis (CTA) – Cognitive tasks required of the user are defined for each traditional task identified in the previous step using cognitive and psychomotor taxonomies. Tasks are gradually decomposed to a satisfactory level.
3) Experimental Validation – A controlled experiment is designed to simulate the task and measure cognitive workload for classified tasks of interest. Experimental and theoretical workload are statistically compared.

This methodology is introduced in the context of its prior application in a companion paper that includes detailed experimental results [18]. The use case scenario is a control room monitoring task from the perspective of the operator, which will be introduced first.

### Use case scenario

A use case scenario is a representation of a real-world process, generalized for the sake of analysis. It is a model of how the parts of a system behave and interact [32]. Developing a scenario is important for CTAWC because it sets the groundwork to cognitively decompose activities and serves as a guide for experimentation and simulation. The scenario specific to this work is control room operation. Control room operators are required to monitor the status of autonomous and semi-autonomous systems over long periods of time while remaining vigilant to respond or intervene, if necessary. In cases where a response becomes necessary, the operator is required to find and analyze salient information, make a decision regarding that information, and respond, typically through some form of physical (e.g., press a button) or sensory (e.g., voice command) system input. In cases where accuracy and time-sensitivity are critical for effective and safe operation, the workstation environment should be designed to minimize operator cognitive workload and maximize task performance. CTAWC provides a baseline model of workload for the task and opens opportunities to evaluate changes in workload in response to design changes. This application was demonstrated in [18] and will be used to support explanations of methodological steps in subsequent sections.

### Procedural task analysis

The original and simplest form of task analysis is called sequential, or procedural task analysis (PTA) [33]. This is the first step of CTAWC. As the name implies, tasks are identified and represented in a sequential manner, which defines the process flow. Decision points, where multiple trajectories of a process are formed, are also commonly integrated into the PTA. Typically, tasks outlined in a PTA are physical or motion-based tasks. In other words, tasks describe what the human is “doing”, in contrast to what the human is thinking (an important addition which is introduced in the next section). There are many ways to perform a PTA and the correct way is highly dependent on the application [34]. This section describes just one approach.

The first step of the task analysis is to identify the overarching goal of the task to be analyzed. This defines the outer-most bounds of the analysis and provides a starting place for decomposition. This should be established at the beginning of the CTAWC process with input from key stakeholders of the system being analyzed.

From there, the overarching task can be decomposed into physical sub-tasks and processes. These should describe the physical actions that take place to achieve the end goal of the system. Once again, this should be done with collaboration from stakeholders who have first-hand experience operating in the system of interest. The process is iterative and can be approached as a continuous, open discussion on whether the tasks identified reflect the true nature of the system. This process is referred to as requirements elicitation.

**Fig. 1:**
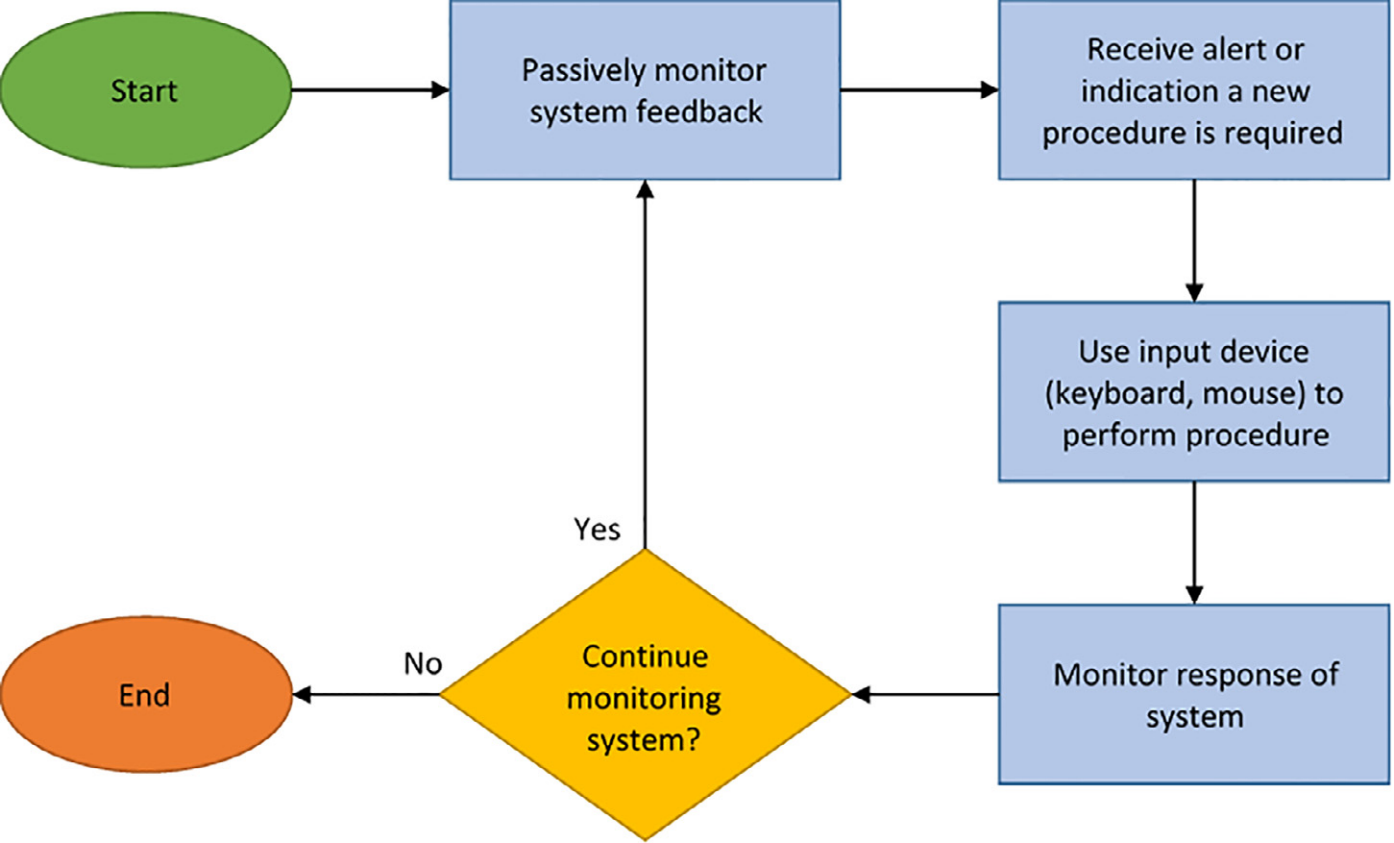
Procedural task analysis for control room monitoring task.

For the control room monitoring scenario, project sponsors from the Naval Air Systems Command (NAVAIR) served as key stakeholders in the requirements elicitation process. A continuous dialog with these key personnel helped to identify the core physical processes necessary for the monitoring task and produced the following basic process flow applicable to most control room operations: 1) Operator passively monitors feedback from the system; 2) Operator receives an alert or indication that some new procedure must be performed; 3) Operator uses input devices (keyboard, mouse) to perform procedures; and 4) Operator monitors the response of the system. A graphical version can be seen in Fig. 1.

### Cognitive task analysis

CTA uses the basic process flow from the prior step as input and identifies the human cognitive actions required to perform all steps of the task. This involves hierarchically decomposing the task into cognitive actions and may involve filling in gaps between tasks as well.

#### Decomposition structure

The structure of this decomposition follows a hierarchical task analysis format [35,36], which provides additional depth for each high-level task with a plan to dictate how to traverse the levels. This structure and the associated plan can account for both concurrent and serial tasks, representing parallel and sequential cognitive processing, respectively.

The scope of cognitive actions should include low-level processing such as recalling basic facts and ideas up to high-level processing such as evaluating criteria and decision-making. Both observable and non-observable tasks can be included. Observable tasks are primarily those in the psychomotor domain related to movement and coordination of the body. Non-observable tasks are memory, decision-making, and sensory processes. Specifics of sub-tasks should be tailored for the use case scenario.

Tasks can be written in many formats. Often a tabular or bulleted list is used where each sub-bullet level represents a level of the decomposition. If special instructions are required for performance of tasks, such as the order, this can be interspersed at the end of levels. For example:

**Table:** 

0. Overarching Task (Goal)
1. Task 1, level 1
1.1. Sub-task 1.1, level 2
1.2. Sub-task 1.2, level 2
1.3. …
*Plan 1: Do 1.1, 1.2, 1.3, … in that order*
2. Task 2, level 1
2.1. Sub-task 2.1, level 2
2.2. Sub-task 2.2, level 2
2.3. …
*Plan 2: Do 2.1, 2.2, 2.3, … simultaneously*
3. Task 3, level 1
…

A graphical or flowchart representation may be desirable in some cases. If the relationships between tasks or the order that tasks are performed is particularly complicated, it may be easier to represent the task analysis as a flow chart. Further, stakeholders who are not familiar with task analysis may find a graphical presentation more intuitive or easier to understand.

#### Evaluating decomposition depth

A common question that arises when performing task analysis is how deep to go with the decomposition. If the decomposition isn't deep enough, then tasks may be too broad and difficult to precisely evaluate. If the decomposition is too deep, there is risk of the analysis exploding into too many variables and task evaluation becoming cumbersome. Additionally, if the goal is to empirically evaluate decomposed tasks, tasks that are too granular may be difficult to isolate/observe in practice. Of the two, it is better to err on the side of additional depth as one can always return to the prior level of depth.

More importantly, the appropriate depth depends on the goals of the analysis. If the researchers have a hypothesis defined prior to the analysis, then the tasks should be decomposed to the point such that the hypothesis can be tested. For example, returning to the control room scenario, if a researcher wanted to test whether control room operators experience elevated cognitive workload when searching for a button to respond to an alarm, then the analysis should be at least deep enough to isolate that individual visual search task.

#### Defining cognitive actions and task syntax

To facilitate the identification of cognitive actions for each previously identified process, cognitive and psychomotor taxonomies can be used. One of the novel contributions of this methodology is the integration of taxonomy-driven classifications of cognitive tasks to model operator cognitive workload. CTAWC utilizes Bloom's taxonomy of the cognitive domain [37] and Harrow's taxonomy of the psychomotor domain [38] to do this modeling. Bloom's taxonomy is a six-tiered model for classification of cognitive skills and is described in Table 1, where each tier corresponds to increased cognitive complexity, thus providing a structure to identify tasks of increasing workload. Likewise, Harrow's taxonomy is a six-tiered model for classification of psychomotor skills and is described in Table 2.

**Table 1:** Bloom's Taxonomy listed in order of increasing cognitive complexity.

	Taxonomy Level	Description	Example Verbs
**1**	Knowledge	Recall of specific facts or ideas.	remember, define, list, memorize
**2**	Comprehension	Understanding and interpreting facts and ideas.	classify, explain, discuss, identify
**3**	Application	The use of prior knowledge in novel situations.	execute, solve, operate, respond
**4**	Analysis	Decomposing a system into its composite parts and examining those parts.	compare, associate, contrast, test
**5**	Synthesis	Combining independent elements to form a new system.	assemble, design, integrate, produce
**6**	Evaluation	Judging the value of a system based on evidence and certain criteria.	judge, appraise, defend, critique

**Table 2:** Harrow's Taxonomy listed in order of increasing psychomotor complexity.

	Taxonomy Level	Description	Example Verbs
**1**	Reflexive Movements	Involuntary movements evoked in response to some stimuli.	flex, extend, stretch, react
**2**	Fundamental Movements	Basic movement patterns which build on reflexive movements.	reach, grasp, walk, jump, crawl
**3**	Perceptual Abilities	Ability to receive information about oneself and the world via one of several sensory systems (vision, hearing, etc.).	sense, perceive, hear, see, feel
**4**	Physical Abilities	The functional characteristics of the body which govern the efficiency of skills in the psychomotor domain.	exert endurance, exert strength, exert flexibility
**5**	Skilled Movements	Complex movement skills which require learning.	dance, drive, juggle
**6**	Non-Discursive Communication	Learned movements and gestures used for communication.	express, posture, gesture

This methodology asserts that these taxonomy classifications can be used to identify and predict workload experienced by operators during cognitive tasks. Bloom's taxonomy primarily addresses non-observable actions, starting at the lowest level of cognitive function in memory-based tasks (Knowledge). As the levels increase, the conscious control required to execute the task also increases, with each higher level composed of the lower level tasks. For example, to perform an Application task, one must first remember specific facts or ideas (Knowledge), understand the recalled information (Comprehension), and then apply it to a novel situation. Harrow's taxonomy, focuses primarily on observable tasks, moving from lower levels of complexity (Reflexive Movements) to higher levels (Non-Discursive Communication). Harrow's taxonomy also considers non-observable sensory tasks (Perceptual Abilities). Combined, Bloom's and Harrow's taxonomy provide a comprehensive categorization of human tasks that can be used to understand how cognitive workload and complexity is represented in a series of actions. The taxonomies also provide a list of verbs (Tables 1 and 2) that can be used, in addition to their synonyms, to develop action words corresponding to each cognitive task.

While originally intended for and most commonly applied to evaluating educational objectives [39,40], CTAWC integrates Bloom's and Harrow's taxonomy into a task analysis framework for assessing operator cognitive workload. There are few past examples of using these taxonomies in conjunction with task analysis [29,41], and to our knowledge there have been no attempts to connect the taxonomy levels to cognitive workload through empirical data.

Returning to the procedural tasks identified prior, one can begin to decompose tasks into cognitive tasks, using Bloom's and Harrow's taxonomy as a guide. A portion of the control room monitoring process is shown in Table 3 for a single task block of the original PTA. The process moves from left to right in the table.

**Table 3:** CTA for PTA task “Receive alert or indication a new procedure is required”.

Procedural Task	Cognitive Task Level 1	Cognitive Task Level 2	Cognitive Task Level 3	Taxonomy Classification
Receive alert or indication a new procedure is required	1. Detect visual alerts	1.1. Perceive visual signal	–	Perceptual Ability
		1.2. Segment field based on visual characteristics	–	Reflexive Movements
		1.3. Identify salient signal	–	Comprehension
		1.4. Identify patterns from salient visual characteristics	–	Comprehension
		1.5. Retain patterns in working memory	–	Knowledge
	2. Detect auditory alerts	2.1. Perceive auditory signal	–	Perceptual Ability
		2.2. Segment field based on auditory characteristics	–	Reflexive Movements
		2.3. Identify patterns from salient auditory characteristics	–	Comprehension
		2.4. Discriminate between multiple auditory signals	2.4.1. Recall prior information on auditory feature discrimination	Knowledge
			2.4.2. Identify salient voice signal	Comprehension
			2.4.3. Identify salient beep signal	Comprehension
		2.5. Retain patterns in working memory	–	Knowledge
	3. Detect tactile alerts	3.1. Perceive vibrotactile signal	–	Perceptual Ability
		3.2. Identify vibrotactile signal	–	Comprehension
		3.3. Retain patterns in working memory	–	Knowledge

In the best-case scenario, cognitive tasks will fit neatly into a single taxonomy level. If a task does not fit into a single taxonomy level, this may indicate a need to decompose tasks further. This process can be used as an additional approach to determine if the task analysis reached an adequate depth. If a task cannot be classified neatly into a taxonomy level, and one does not wish to decompose tasks any further, then there are a few other options. Multiple taxonomy levels can be matched to individual tasks, however this may complicate the model and hinder interpretation. Another approach is to use the highest taxonomy level (highest level of cognitive complexity) applicable to the task, the logic being that the operator will experience cognitive workload at least as much as that taxonomy level implies. For example, if a task requires the use of comprehension and evaluation then, one could assume, that the operator will experience cognitive workload at the level evaluation tasks typically generate. This approach assumes that there isn't an interacting effect between multiple taxonomy levels occurring, which may not always be the case in all scenarios.

### Experimental validation protocol

After completing the CTA, an empirical study can be designed to validate the decomposition. This section discusses a protocol for designing a study to validate the assumed hierarchy of task complexity based on Bloom's and Harrow's taxonomy.

#### Experimental research questions

The first task is to define experimental questions based on the decomposed tasks and the goals of the research. In general, the objective will be to investigate differences in performance between tasks. Determining what tasks to investigate and the level of granularity to define hypotheses is the initial challenge. This will depend on resource availability, as constraints will limit the number of hypotheses that can be feasibility tested. Also important are the goals of the analysis. If specific tasks are more important to investigate than others, then those should be prioritized and targeted.

Depending on the complexity of the overarching tasks, there may be many more tasks than can be feasibly tested in a laboratory experiment due to resource and recruitment burden. In the companion paper [18], the lowest-level of the decomposition resulted in 29 individual tasks, which is too many to control in an experiment. There are several practical considerations related to the study length and resources. Experimental run time is a critical metric, with subject recruitment rate having a strong relationship to the amount of time the subject must devote to the experiment. Additionally, it is problematic from a safety and ethics perspective to keep subjects in an experimental facility for more than 90 min without a break, which compromises continuous biosensing and calibration of equipment. Resources such as wireless devices and other equipment may have limitations for run time, requiring power source and system memory replacements mid-experiment. For the control room study, the target population consisted of University students, therefore timing was a critical factor, as many students have scheduling limitations.

To address the previous resource limitations, instead of trying to measure each individual task, one could opt to move up a level in the decomposition and create a hypothesis around all the classifications applied to each task on that level. The full CTA for the control room study can be seen in Table 4. It was hypothesized that the tasks classified with the highest taxonomy levels will result in the highest levels of cognitive workload, which aligned with experimental evidence [18]. The resulting task model was a sequence of four tasks, where Task 2 (corresponding to Task 4 in Table 4) and Task 3 (corresponding to Task 5 in Table 4) contained Synthesis and Analysis from Bloom's taxonomy and were hypothesized to be higher workload tasks. Task 1 (corresponding to Tasks 1–3 in Table 4) and Task 4 (corresponding to Tasks 6–7 in Table 4) only contained low-level tasks from Bloom's taxonomy and tasks from Harrow's taxonomy, and it was therefore hypothesized that operators would experience less cognitive workload during these tasks. Therefore, the resulting hypothesis was to test whether there was a significant difference in operator performance and cognitive workload between each of the 4 tasks.

**Table 4:** Resulting cognitive task analysis originally developed in [16].

Tasks	Taxonomy Level
**0. Monitor UAV system**	
**1. Detect visual alerts**	
1.1 Perceive visual signal	Perceptual Ability
1.2 Segment field based on visual characteristics	Reflexive Movements
1.3 Identify salient signal	Comprehension
1.4 Identify patterns from salient visual characteristics	Comprehension
1.5 Retain patterns in working memory	Knowledge
**2. Detect auditory alerts**	
2.1 Perceive auditory signal	Perceptual Ability
2.2 Segment field based on auditory characteristics	Reflexive Movements
2.3 Identify patterns of salient auditory signals	Comprehension
2.4 Discriminate between multiple auditory signals	
2.4.1 Recall prior information on auditory feature discrimination	Knowledge
2.4.2 Identify salient voice signal	Comprehension
2.4.3 Identify salient beep signal	Comprehension
2.5 Retain patterns in working memory	Knowledge
**3. Detect tactile alerts**	
3.1 Perceive vibrotactile signal	Perceptual Ability
3.2 Identify vibrotactile signal	Comprehension
3.3 Retain patterns in working memory	Knowledge
**4. Identify location of critical/non-critical alert (sensory integration)**	
4.1 Integrate all sensory signals	Synthesis
4.2 Perceive auditory signal location	Perceptual Ability
4.3 Perceive visual signal location	Perceptual Ability
4.4 Identify direction of auditory salient signal	Reflex Movements
4.5 Identify screen location of visually salient signal	Reflex Movements
**5. Analyze alert category**	
5.1 Recall prior information/training on features differentiating alerts	Knowledge
5.2 Associate salient signal alert category with prior information	Analysis
5.3 Identify alert category	Comprehension
**6. Respond to non-critical alert**	
6.1 Recall prior information on procedural response to non-critical alert	Knowledge
6.2 Acknowledge non-critical alert	Fundamental Movement
6.3 Type textual information on critical alert response	Fundamental Movement
**7. Respond to critical alert**	
7.1 Recall prior information on procedural response to critical alert	Knowledge
7.2 Acknowledge critical alert	Fundamental Movement
7.3 Type textual information on critical alert response	Fundamental Movement

One could take an alternative perspective and make a hypothesis about the interaction or cumulative effect of multiple taxonomy levels occurring during the same task. This was not the approach taken in the accompanying work [18], but there is room for future work investigating this idea. In either case, an approach for empirically validating these hypotheses is discussed in the next sections. Discussed first are validation metrics for human performance and cognitive workload.

#### Human performance measures

Experimental validation of taxonomy classifications requires performance metrics. Objective and subjective measures of human performance can be utilized to encourage study robustness through a mixed method experimental approach. Objective metrics can measure workload and performance directly using accuracy or timing, and indirectly with biosensors for neurophysiological data correlated with workload. Subjective metrics use participant feedback to directly quantify perceptions of workload. This method suggests the use of both to validate task workload classifications for a more robust study. Discussed next are the criterion used to select the objective and subjective measures for the experimental validation study.

For objective, indirect measures of human performance, there are several factors to consider when selecting the biosensing hardware. Most biosensors are wearables and may be influenced by movement of the wearer. Body movement can cause issues with data quality in biosensors, such as with electroencephalography (EEG) headsets [42], [43], [44]. In these cases, additional efforts to filter movement artifacts may be required [45]. Biosensors may also interfere with the wearers ability to perform a task. As well as being susceptible to motion artifacts, biosensors such as a pulse oximeter worn on the finger can make it difficult for subjects to physically interact with a system using their hands [46]. The selected biosensors should not inhibit the subject's ability to move and complete the tasks necessary for the experiment.

Another factor to consider when choosing a biosensor is whether to buy a commercial off the self (COTS) product or to custom build a system. Purchasing a COTS biosensor may provide the opportunity to review other users’ experiences, come with software to assist with data collection and processing, and provide access to technical support. Downsides of a purchased biosensor include limited control over the data and cross-manufacturer software integration difficulties. Proprietary software may perform data calculations that cannot be altered or examined due to intellectual property restrictions. Building a custom biosensor bypasses these limitations but requires additional development, debugging, system integration, and validation time. A lab-built biosensor requires research into which parts to use and should be benchmarked against commercial biosensors to test accuracy and reliability. Depending on time constraints, this may be infeasible.

For the control room study, subjects were required to turn their heads to view multiple screens and type responses to cues on those screens. This eliminated the use of biosensors whose data quality is significantly impacted by subject motion. The need for subjects to type also disqualified the use of biosensors that attach to the hand. Ultimately, pupillometry via COTS biosensors (SMI VR ETG eye-tracking glasses) (Fig. 3A) was selected to indirectly measure subject workload. Additional detail on the eye tracking system setup is provided in Section 2.5.2. Pupil response has been demonstrated as a reliable indirect measure of cognitive workload [47], [48], [49]. Further, these sensors are designed to allow the wearer to move their head and minimally influence the data.

Subjective feedback from participants can reinforce objective data and provide direct insights about a system operator's experience. For workload assessment, pre-validated instruments such as the Subjective Workload Assessment Technique (SWAT) [50] and NASA Task Load Index (NASA-TLX) [51] can be applied to many simulations. These generalized assessment tools have the advantage of being well validated across many domains, however, they may not meet the time constraints of the experiment or provide the context specificity desired. For the control room study, participant feedback was required at several intervals within the simulation. This required participants to be able to provide feedback quickly, with minimal disruption to the flow of the study. These requirements were not satisfied by the existing survey instruments. For this reason, a custom survey was developed. This provides the dual benefit of being able to design the survey to the needed length and tailor questions to specifically evaluate each task of interest.

The medium for administering the survey should be considered as well. Paper and pencil are likely the most reliable medium, however, this requires handoffs between researchers and participants and introduces manual administrative and data input tasks. If the simulation requires interaction with a computer, then a digital survey format is advantageous. For the control room study, participants were already interacting with a screen, therefore the survey was implemented with a batch file code that could be executed remotely.

### Experimental setup

There are many logistical considerations when designing a human performance simulation. This section discusses how the study requirements were carefully integrated within the limitations of the hardware and software available.

#### Simulation requirements

**Fig. 2:**
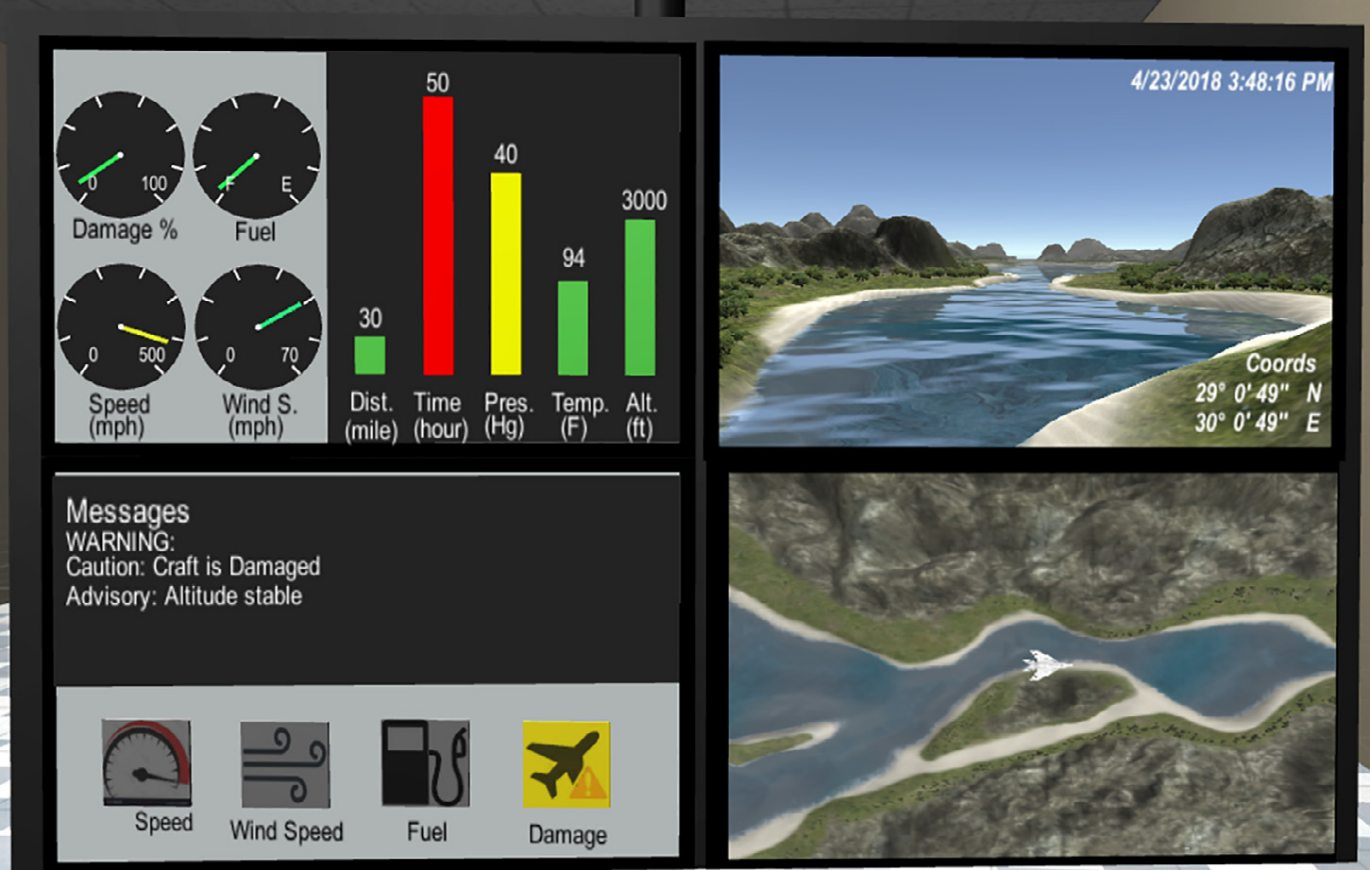
Finalized four quadrant layout used in the simulation. Screens display the following information: 1) health of the UAV (see through dials/bar graphs); 2) communications system (through which the participant can issue commands); and both a 3) forward and a 4) downward facing terrain camera.

Scenario development was conducted with thorough planning and input from several sources. Stakeholders were interviewed, and a literature review on control room design was performed to ensure the simulation tasks and environment aligned with the real-world tasks. Stakeholder feedback is an important aspect of the iterative design process and confirms that what is being designed conforms to stakeholder expectation [52], [53], [54], [55]. Feedback was iteratively gathered from the project stakeholders to ensure simulation realism. A primary goal of the screen layout, communicated by project stakeholders, was to be generalizable to many monitoring scenarios. Therefore, the replication of a specific monitoring and control software was avoided. In addition, several critical systems had to be simultaneously accessible to the operator. These requirements led to the development of a quadrant layout providing information on system health, location, real time navigation (front facing camera), and communication (Fig. 2). A mock-up of the control room was presented to project sponsors from the NAVAIR Human Systems Performance Division. The sponsors were asked to provide feedback on simulation features, including the number of screens that should be included and the realism of displayed information.

In addition to stakeholder input, control room and design standards, scholarly literature, and existing control room images were reviewed to help improve the accuracy of the control room design. Feedback in simulated environments should be designed carefully to elicit realistic responses from participants. In the control room simulation, the form of the multimodal sensory feedback relied heavily on a review of these sources. Department of Defense standard MIL-STD-1472F [56], Department of Transportation standard DOT HS-812–360 [57], and American National Standards Institute standard ASTM F116–95a [58] were used to inform the design of visual, audio, and tactile feedback, respectively. Visual, tactile, and audio alarms are used in a variety of safety critical systems including control rooms [59,60], automobiles [57,^61^,62], and ships [63]. Visual alarm cues can involve icons appearing on the screen, changing colors [58], and flashing [56]. For visual cues in this experiment, the colors yellow and red were used to denote non-critical or critical alarms, respectively. Several standards recommend the use of the color red to gain an individual's attention in an emergency and yellow as a cautionary warning [56,^58^,64].

Tactile feedback was provided to the participants using a custom vibrotactile wristband (Fig. 4, 3B). The vibrotactile bracelet was composed of an Arduino Mini, a mini vibrating disc motor, a small solderable breadboard, a 3D printed case, and a sweat wristband. Arduino hardware was used due to the flexibility of customization of the hardware and code. The Arduino Mini was used to keep the wrist bracelet small and lessen the chance of it interfering with subject movement. A sweat band was picked as the wearable because it would be able to stretch to fit on a variety of wrist sizes and make the donning/doffing process easier. Tactile feedback was used due to its presence in everyday objects such as cell phones and in automobile and plane safety systems. The feedback frequency used was around 180 Hz to 200 Hz since it is a similar range used in cell phones [65,66]. The tactile feedback was applied for milliseconds at a time to get an individual's attention [57,^62^,^67^,68]. This form of feedback is unique because it uses a sensory channel that is not already being used to observe the environment. Sense of sight and sound can be overwhelmed in monitoring tasks because there are multiple pieces of information competing for attention.

**Fig. 3:**
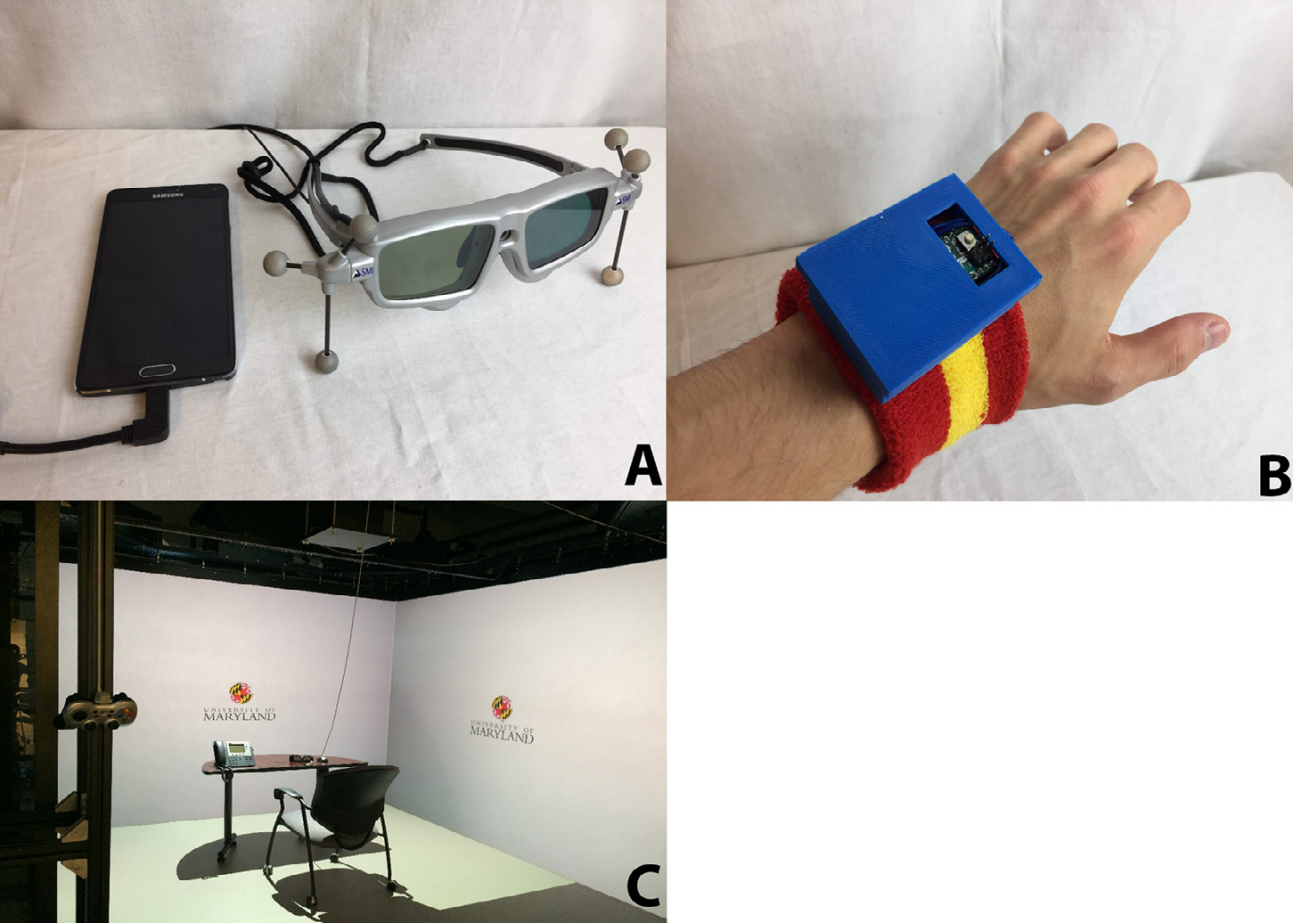
Hardware used during the study. A. SMI eye tracking glasses B. Lab built vibrotactile bracelet C. VR CAVE system.

**Fig. 4:**
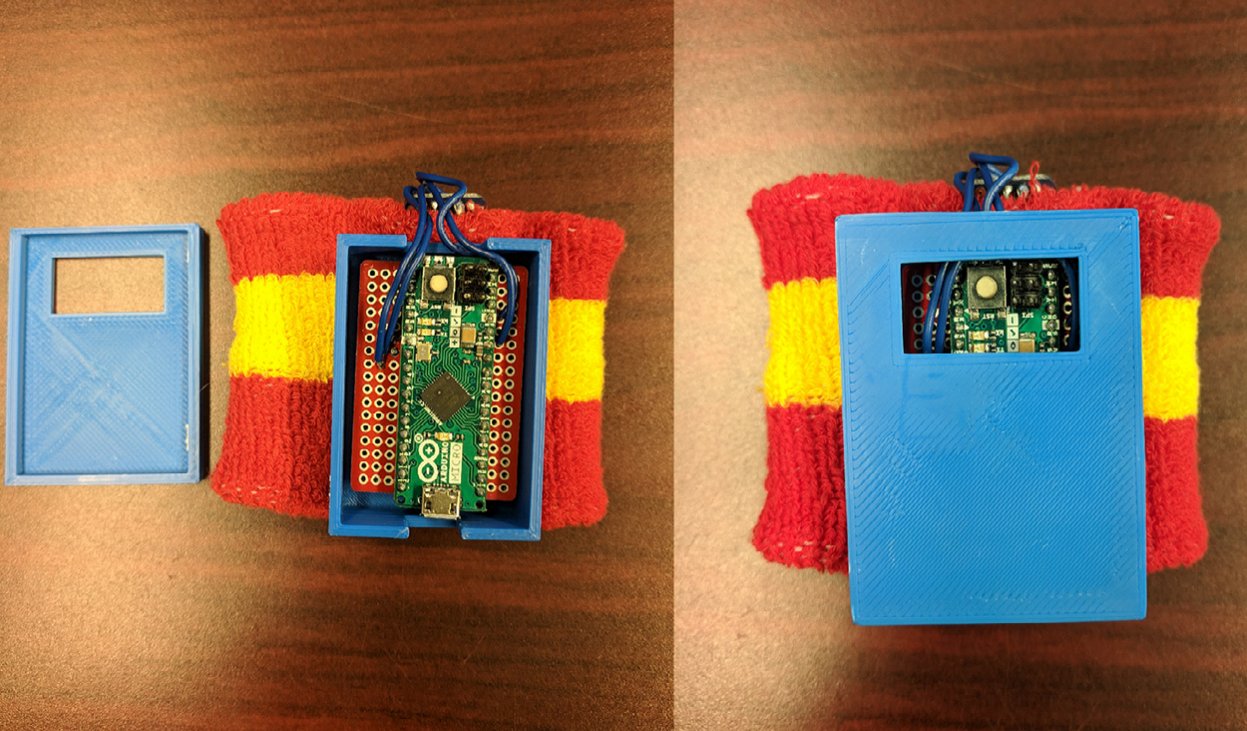
Vibrotactile bracelet built for the experiment. A. An inside view of the components B. Final product.

Two types of audio alarms were used in the study: beep and voice. Both were designed to be loud enough to be heard over the ambient noise of the Cave Automatic Virtual Environment (CAVE) system running the experiment, and to utilize the surround sound system. The beep was designed to be distinct to not get lost in background noise programmed into the experiment [69]. Voice cues were used because they have been shown to improve a subjects ability to respond accurately [70,71]. The voice used in the experiment was that of a monotone woman. Evidence has shown that a monotone voice can increase response time when used in alerts [58,72]. It is widely agreed that the voice should be mature and that the message should be succinct, relay the criticality of the alarm through tone or word choice, and be repeated multiple times [56,^58^,64].

#### Hardware setup

**Fig. 5:**
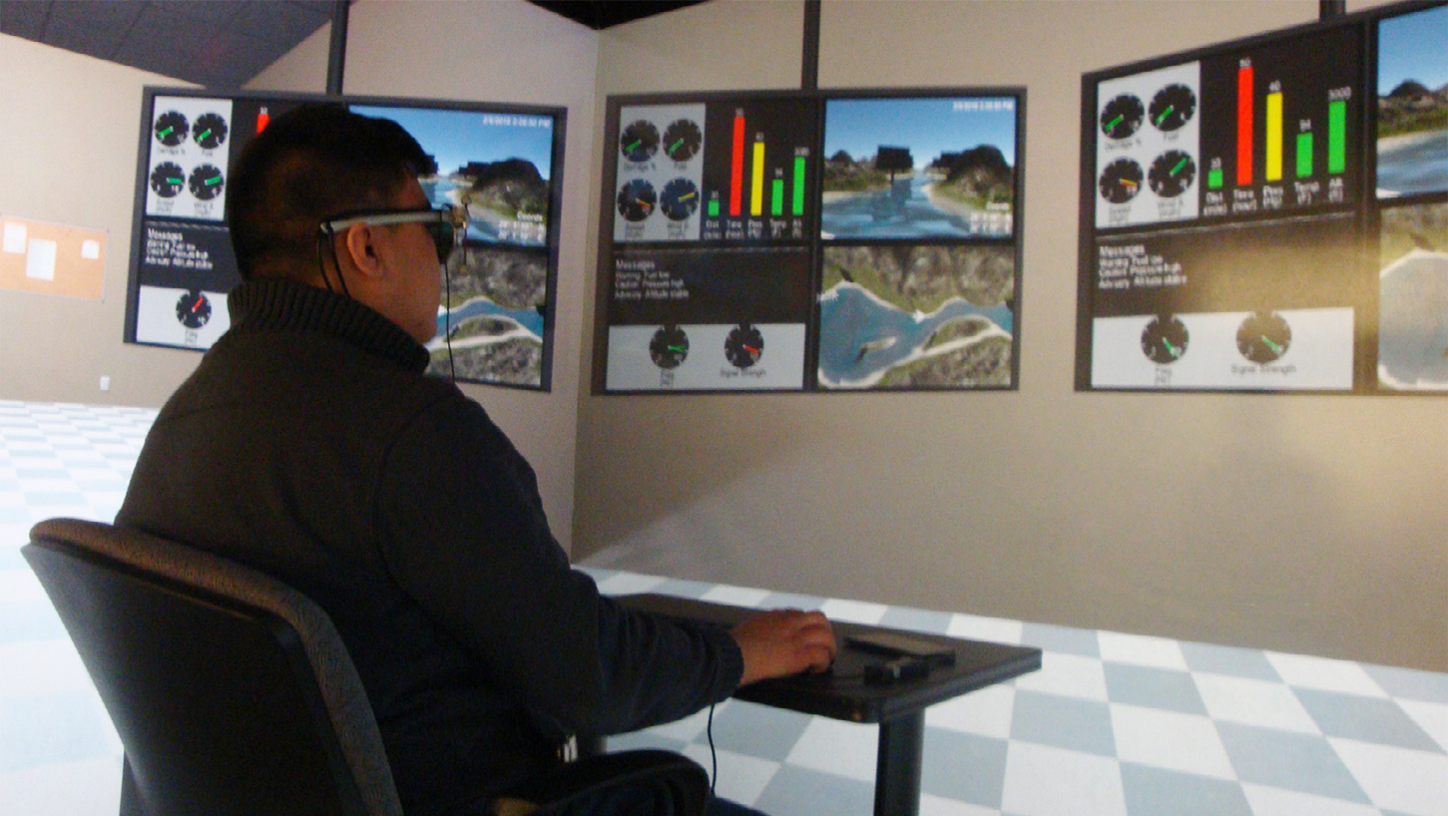
Participant operating the UAV simulation.

In the companion paper [18], a simulation was built in a virtual reality (VR) CAVE (Fig. 3C) consisting of three walls on which simulations can be projected by Barco Galaxy 6 Classic+ projectors. It is possible that this simulation could have been built for a head-mounted display (HMD), however the use of the CAVE environment allowed participants to interact with other physical artifacts, such as a keyboard for simulation input, that can provide additional immersion. Additionally, it does not require donning of additional hardware, which an HMD requires. Six Tannoy system 600 speakers provided surround sound for audio feedback. The simulation was intended to replicate a UAV control room. Advanced Realtime Tracking (ART) motion capture cameras, consisting of 4 cameras placed in the 4 corners of the CAVE, and Dtrack software were used to track the subjects head in space and monitor the screens they were looking at during the simulation. Six motion capture markers on the side of the SMI eye tracking glasses, 3 markers per side, were used to create a rigid body that Dtrack can use to calculate the subject's head position throughout the experiment. Fig. 5 shows a participant engaged in the simulation with equipment donned.

In addition to the VR and tracking infrastructure, several elements were considered for data acquisition. The hardware set-up should be optimized to minimally impact the simulation. This includes the advanced charging of electronics. Back-up equipment and batteries should be available when possible. A standardized and repeatable procedure for setting up equipment is critical to be established and practiced prior to running subjects. This will help minimize the time required for set-up and will help to eliminate variability between participants. In the control room simulation, participants were required to don several pieces of equipment, including eye-tracking glasses and a vibrotactile bracelet. Participants were aided as much as possible when donning the equipment and given explicit instruction when direct aid could not be given.

**Fig. 6:**
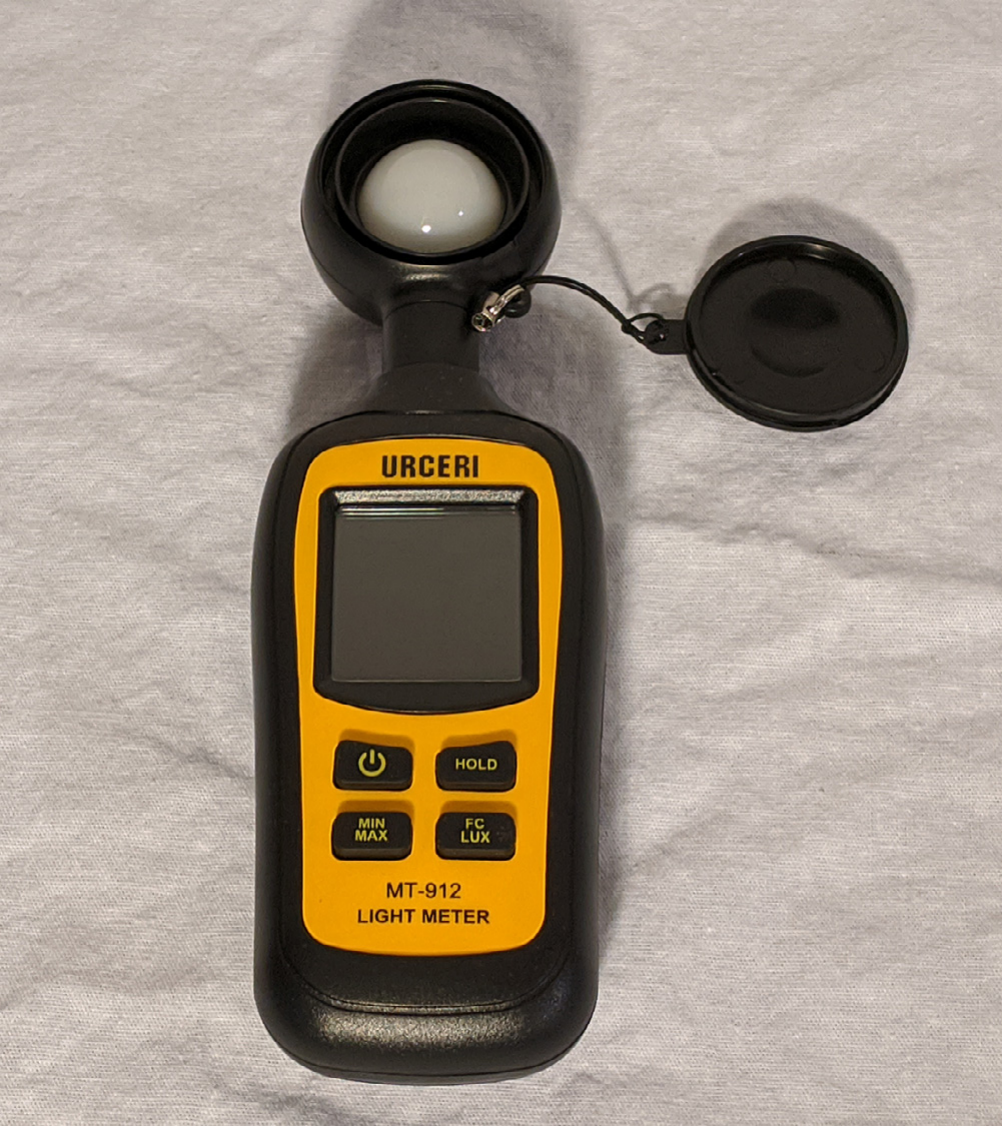
Urceri MT-912 light meter used for luminance testing.

For eye-tracking hardware, it should be verified that ambient light and light from the testing or simulation environment have minimal impact on pupil response. Ambient lighting should not be changed throughout the experiment, or between experiments. If lighting in the experimental set-up must change, then a light meter should be used to verify that the change in luminance is minimal. If there are significant changes in luminance throughout the experiment, this could confound pupil response results. For the control room experiment, luminance testing was performed with the Urceri MT-912 Light Meter (Fig. 6). This was necessary to verify that lighting changes from the CAVE simulation would not influence pupil response. To do this, the maximum and minimum lighting conditions for the simulation were identified. Luminance measurements were taken at eye level height, facing the center and 4 corners of each CAVE screen under each condition. The measured luminance during each condition was virtually identical, and it was concluded that changes in lighting during the simulation should have minimal influence on participant pupil response.

The experimental layout is also critical. If wireless electronics can be used, this is ideal because it minimizes the likelihood of tripping and equipment accidently becoming disconnected, as well as providing a non-clustered research environment. This said, if possible, wired back-ups should be available. In practice, wireless connectivity is not always as reliable as hardwired equipment, which is particularly critical when data is being logged in the millisecond range. Reliability issues with a wireless keyboard were encountered during the control room study that required the substitution of a wired keyboard.

#### Software development

Unity software was used to create the simulation. Specifically, Unity 5.3.5 was used for software compatibility with getTeal3D, a Mechdyne CAVE rendering software. A review of control room images revealed that control rooms often use a quadrant-based layout to display information. The 3D objects used to create the four screens were created from scratch or from assets that could be found in the Unity store.

**Fig. 7:**
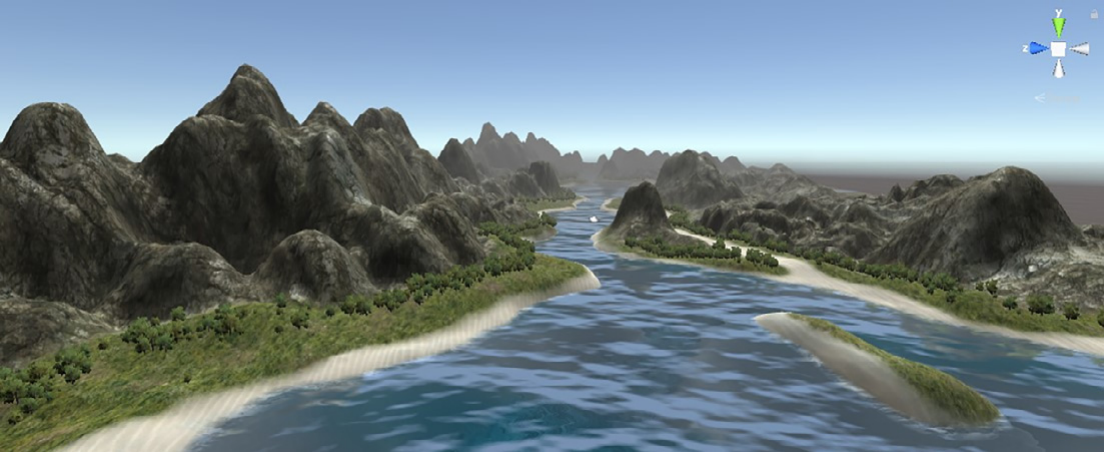
Sample of simulation terrain.

The simulation monitors were composed of a rectangular prism with interactive objects overlaid. The terrain that the UAVs flew over was created with a terrain tool built in Unity (Fig. 7). The UAV asset was imported from the Unity asset store. Two camera views were used to show the UAV flying over the terrain; one attached to the front of the UAV and one looking down on the UAV. Both cameras were set to move with the UAV as it moved over the various terrains. A short video of the UAV flying through the terrains was captured and embedded in the simulated monitor. The dials on the health screen reflected the health of the UAV throughout the experiment and was designed to change values in response to the alarm status. For example, if the critical fuel alarm was triggered, then the fuel dial would move to empty.

Communications was the only screen that allowed direct subject interaction. Information gathered from the standards documents were used to guide the presentation of information. The communications screen changed color to attract the subject's attention when an alarm was triggered [56]. As mentioned earlier, the colors yellow and red were used to denote non-critical or critical alarms per standards documentation [56,^58^,64]. If the subject was running a version of the alarm scenario that required tactile feedback, then the vibrotactile bracelet would activate when the communications screen lit up. If the subject was running the voice audio version of the scenario, then they would hear a monotone, female voice stating which alarm was triggered (e.g., “Low fuel”). Ambient office sounds were imported into the simulation to distract participants and further enhance simulation realism. Office sounds were recorded in a noisy office where machines and background speech can be heard.

**Fig. 8:**
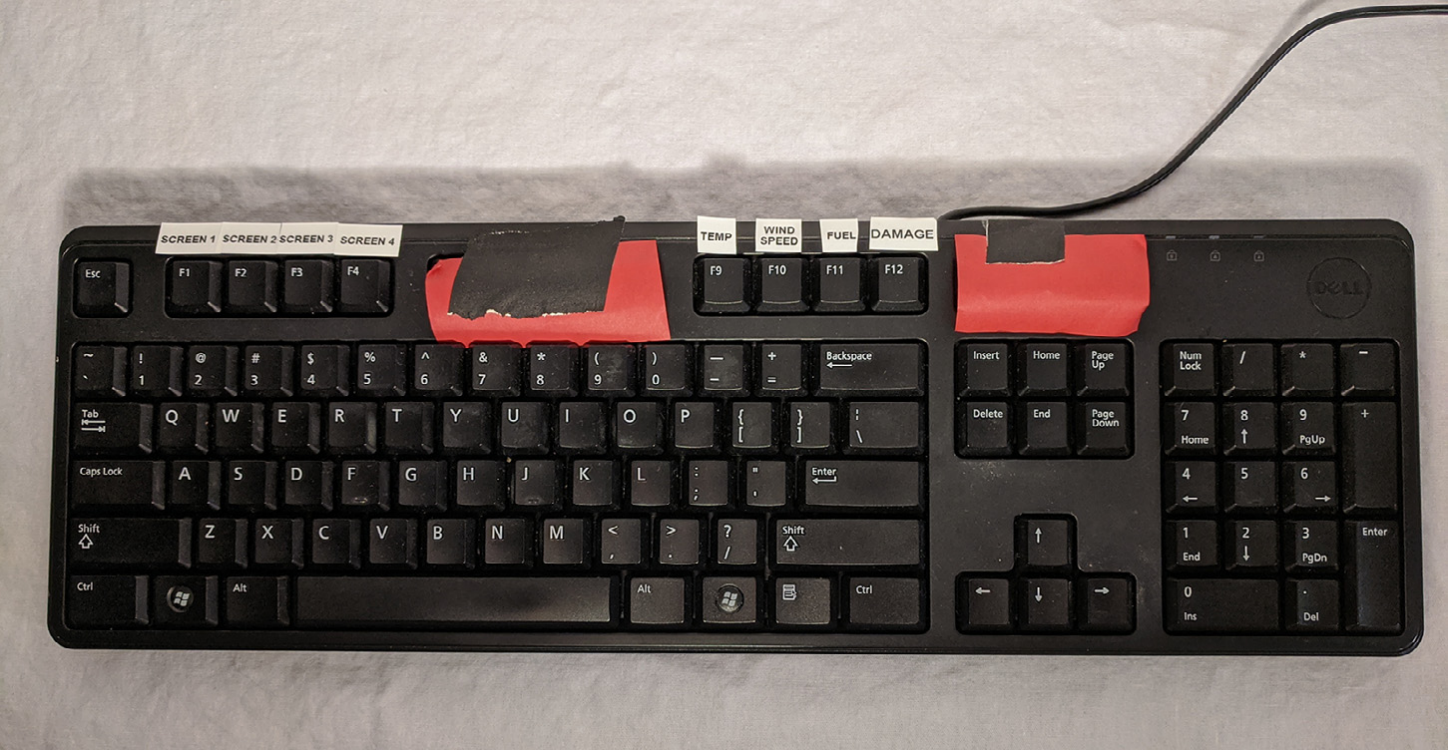
The keyboard layout for simulation command input.

External devices were programmed into the simulation when possible. The SMI glasses were not integrated into the simulation software due to the device being controlled by a secondary device; a Samsung Galaxy Note 4. For user input and response, a standard QWERTY keyboard was used. It was selected because tactile keyboards are commonly used for a multitude of tasks in modern UAV control rooms. Additionally, the keyboard was selected as the main input device because it provided a simple, familiar, and instantaneous medium for input. Keyboard input was not subjected to the frame rate drops and the lag the simulation might encounter. Subjects could select the desired screen (1–4) using the F1-F4 keys (Fig. 8). Once subjects selected the correct screen, keys F9-F12 could be used to select which alarm to acknowledge. F-keys not utilized as part of the experiment were occluded from subject view to minimize mistyping. The communications screen then would ask the user if further action was required. Subjects were required to type “0” for non-critical alarms or “1” for critical alarms and press the “Enter” key to log their response.

#### Training material

**Fig. 9:**
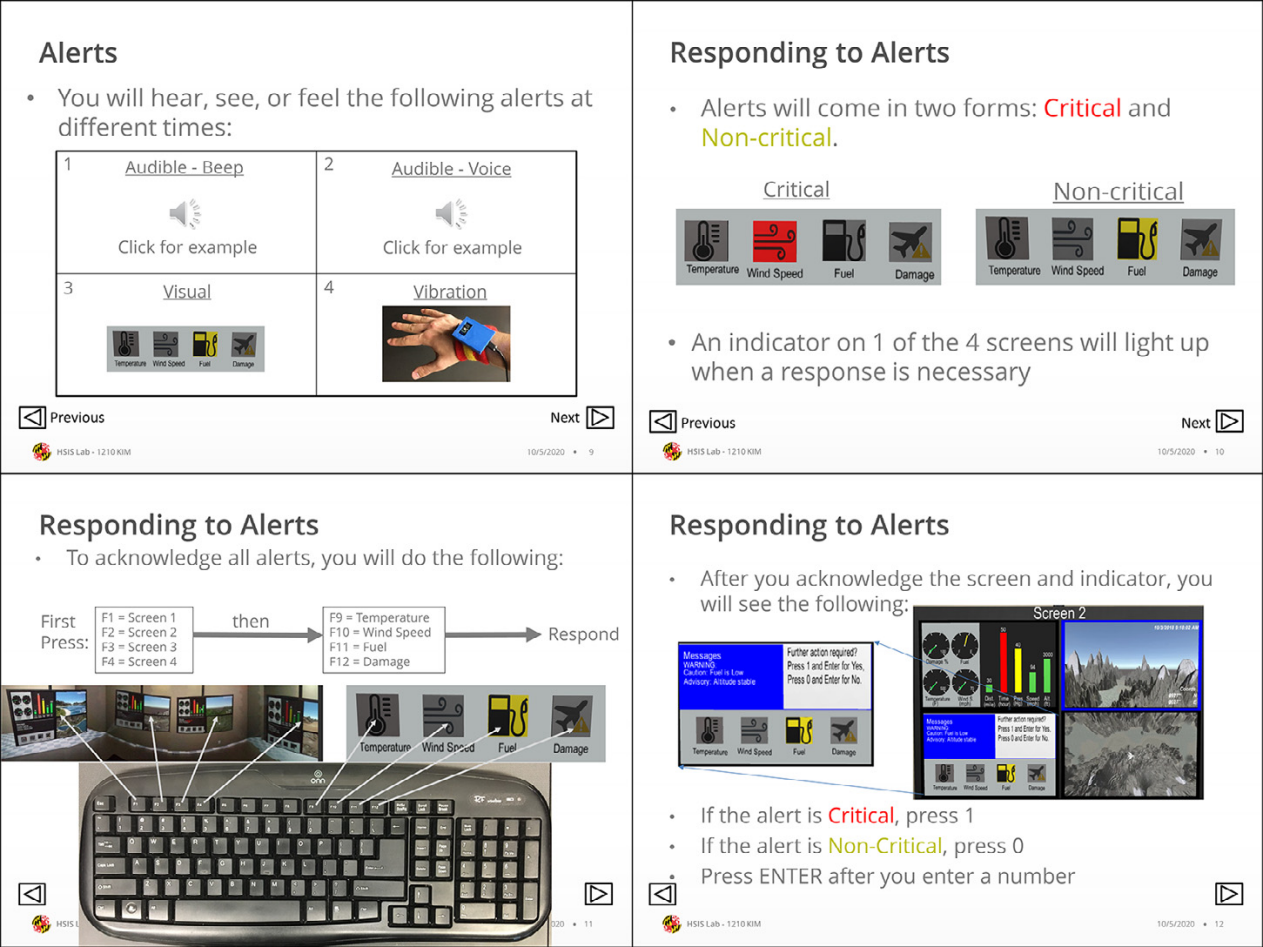
Sample of participant training slides.

Prior to performing the simulation, it was necessary for each participant to be trained on the scenario and simulation tasks they would perform. A slide deck was developed to facilitate training (Fig. 9). The training material included a brief background on the context of the scenario. This was kept brief to not overwhelm participants with information irrelevant to performing the tasks at hand. Details were provided about the sensors and equipment to be worn, as well as steps that can be taken by participants to minimize influence on the sensor data. This included instructions to avoid talking, touching the head or face, and touching the devices. Further information was included regarding the tasks the participants would perform, including an embedded video demonstration.

Prior to the simulation, the participant would be seated in front of an interactive whiteboard with the slide deck loaded. Participants would be instructed to advance through the slides, and to ask the study proctor questions as needed. It is critical for researchers to contribute minimal influence to the training protocol across participants. Differences in training could confound results.

### Data analysis

Procedures for pre-processing and analysis of data are heavily dependent on the performance measures and equipment selected. The steps used for pre-processing simulation data and the tools used for analysis in [18] are briefly discussed here.

#### Preprocessing of data

The bulk of the data preprocessing was performed on eye tracking data. Eye tracking data is known for being noisy and needing substantial cleaning. Corrective procedures can be used to ensure data is minimally influenced by noise and confounding factors. Preprocessing steps for pupil response data included temporally aligning data with the simulation, removing noise, ensuring data fits within known physiological limits, and adjusting data with a baseline correction.

Preprocessing began with aligning the eye tracking data with the simulation start time. Control of the SMI glasses could not be integrated into the simulation, requiring that they be started before the simulation. The simulation was executed as quickly as possible following starting the glasses, but lag would often interfere with the simulation starting time. The recordings of the experiment were manually reviewed to denote how much time passed between starting the glasses and the simulation start.

A MATLAB script was created prior to the study to perform the rest of the preprocessing. Removing noise is a common practice when analyzing data [73], [74], [75]. The removal of noise can minimize unwanted influence due to random fluctuation and confounding factors (e.g. removing pupil data that is outside the biological range) [76]. The code scanned the data and removed data points that were outside of the possible biological range for pupil diameter. Anything less than 2 mm or greater than 8 mm was removed [77]. Any subjects who had more than 50% of their data removed after this was removed from analysis.

Baseline correction of data is used to facilitate comparison between participants and has been shown to correct for random fluctuations in pupil data [74]. Baseline correction is done by taking data collected from a period of rest and subtracting the data to be analyzed by an average value during the rest period. Subtractive, as compared to divisive, baseline correction has been found to be less susceptible to distortions in the data [74]. The baseline data consisted of pupil values from 2 s prior to the first alarm of each simulation.

#### Data analysis tools

To answer the core hypotheses identified in 2.4.1, several statistical analyses were performed. Regardless of the type of performance or workload data collected, this general analysis should be applicable. In the control room simulation, the goal of the analysis was to determine if there was a statistical difference in performance measures for Tasks 1–4. Each task can be thought of as an independent variable, and each performance measure can be treated as a dependent variable. As such, a model can be created for each performance measure. For the control room study, performance measures included pupil response data and survey response data. Pupil response was a continuous variable, therefore linear regression was used. Likewise, survey response was a binary variable, therefore logistic regression was used. As always, it is important to check standard model assumptions required for fitting a linear model.

In the case of [18], four simulations were performed with four task sequences each. It was hypothesized that participant performance may increase as time progresses (i.e. a learning effect). Because simulations and trials were spaced in approximately equal time segments for all participants, each could be treated as a continuous control variable in the models. The continuous assumption may not hold in all cases. If the periods of time are not equally spaced and exact time is not recorded, then it may be better to treat time periods as categorical or ordinal variables.

With categorical independent variables, regression coefficients will only provide effects with respect to the reference category. Multiple comparisons will need to be performed to identify differences between all tasks. As more comparison are made, the probability of finding false positives significantly increases. As such, a correction to the p-value should be applied to reflect a more conservative estimate. Many procedures exist, such as the Tukey test and Bonferroni correction, and most statistical programming packages will have built in functions to perform them [78].

Finally, as an additional step, correlation between performance variables can be investigated. This can provide insight as to whether indirect measures of performance serve as good indicators of direct performance. In these cases, direct measures of performance can be modeled as dependent to indirect measures. By including each task and each indirect performance variable as independent variables, the effect of each indirect measurement can be estimated independent of the task type. This important because each task type will likely have an inherent amount of time needed or accuracy required, independent of participant performance. In [18], some evidence was found to support task time having an association with pupil response and survey response.

## Conclusions

Optimizing a system for maximum human performance requires an understanding of the cognitive tasks required of the user. CTA provides a process for isolating the distinct mental processes performed by system operators. This paper describes an approach to CTA that integrates cognitive and psychomotor taxonomies for predicting cognitive task workload. This approach was demonstrated on a control room monitoring task and demonstrated its ability to discriminate between high and low task complexity. This framework could be potentially applied to any system where unobservable human action plays a significant role in system operation. Future work should include efforts to validate this approach in different settings. Future work should also seek to further demonstrate the discriminating power of the taxonomy classifications. Currently, only “high” and “low” workload was discriminated. Demonstrating that individual levels of the taxonomies can be used to discriminate cognitive workload experienced could provide further validity. Ensuring that systems operate effectively and safely requires models of operator workload to provide a framework for system optimization. This work provides an approach for creating and validating those models.

## Declaration of Competing Interest

The Authors confirm that there are no conflicts of interest.

## References

[1] Clark R., Feldon D., Van Merrienboer J.J.G., Yates K., Early S. (2008). Cognitive task analysis. Handbook of Research on Educational Communications and Technology.

[2] Haapalainen E., Kim S., Forlizzi J.F., Dey A.K. (2010). Psycho-physiological measures for assessing cognitive load. Proceedings of the 12th ACM International Conference on Ubiquitous Computing.

[3] Smith B.P., Browne M., Armstrong T.A., Ferguson S.A. (2016). The accuracy of subjective measures for assessing fatigue related decrements in multi-stressor environments. Saf. Sci..

[4] Dias R.D., Ngo-Howard M.C., Boskovski M.T., Zenati M.A., Yule S.J. (2018). Systematic review of measurement tools to assess surgeons’ intraoperative cognitive workload. BJS.

[5] van Winsum W. (2018). The effects of cognitive and visual workload on peripheral detection in the detection response task. Hum. Fact. J. Hum. Fact. Ergon. Soc..

[6] Lyell D., Magrabi F., Coiera E. (2018). The effect of cognitive load and task complexity on automation bias in electronic prescribing. Hum. Fact. J. Hum. Fact. Ergon. Soc..

[7] Mehler B., Reimer B., Coughlin J., Dusek J. (2009). Impact of incremental increases in cognitive workload on physiological arousal and performance in young adult drivers. Transp. Res. Rec. J. Transp. Res. Board.

[8] Ranchet M., Orlosky J., Morgan J., Qadir S., Akinwuntan A.E., Devos H. (2017). Pupillary response to cognitive workload during saccadic tasks in Parkinson's disease. Behav. Brain Res..

[9] Marquart G., de Winter J. (2015). Workload assessment for mental arithmetic tasks using the task-evoked pupillary response. PeerJ Comput. Sci..

[10] Shakouri M., Ikuma L.H., Aghazadeh F., Nahmens I. (2018). Analysis of the sensitivity of heart rate variability and subjective workload measures in a driving simulator: the case of highway work zones. Int. J. Ind. Ergon..

[11] Di Flumeri G., De Crescenzio F., Berberian B., Ohneiser O., Kramer J., Aricò P., Borghini G., Babiloni F., Bagassi S., Piastra S. (2019). Brain–computer interface-based adaptive automation to prevent out-of-the-loop phenomenon in air traffic controllers dealing with highly automated systems. Front. Hum. Neurosci..

[12] Aricò P., Borghini G., Flumeri G.D., Colosimo A., Graziani I., Imbert J., Granger G., Benhacene R., Terenzi M., Pozzi S., Babiloni F. (2015). Reliability over time of EEG-based mental workload evaluation during air traffic management (ATM) tasks. Proceedings of the 37th Annual International Conference of the IEEE Engineering in Medicine and Biology Society (EMBC).

[13] Kenneth P. Wright J., Hull J.T., Czeisler C.A. (2002). Relationship between alertness, performance, and body temperature in humans. Am. J. Physiol. Regul. Integr. Comp. Physiol..

[14] Young M.S., Brookhuis K.A., Wickens C.D., Hancock P.A. (2015). State of science: mental workload in ergonomics. Ergonomics.

[15] Evans D.C., Fendley M. (2017). A multi-measure approach for connecting cognitive workload and automation. Int. J. Hum. Comput. Stud..

[16] Kuchinsky S.E., Jr K.I.V., Ahlstrom J.B., Cute S.L., Humes L.E., Dubno J.R., Eckert M.A. (2016). Task-related vigilance during word recognition in noise for older adults with hearing loss. Exp. Aging Res..

[17] McIntire L.K., McIntire J.P., McKinley R.A., Goodyear C. (2014). Detection of vigilance performance with pupillometry. Proceedings of the Symposium on Eye Tracking Research and Applications.

[18] Knisely B.M., Joyner J.S., Rutkowski A.M., Wong M., Barksdale S., Hotham H., Kharod K., Vaughn-Cooke M. (2020). A cognitive decomposition to empirically study human performance in control room environments. Int. J. Hum. Comput. Stud..

[19] Moreira M., Peixoto C. (2014). Qualitative task analysis to enhance sports characterization: a surfing case study. J. Hum. Kinet..

[20] Stanton N.A. (2006). Hierarchical task analysis: developments, applications, and extensions. Appl. Ergon..

[21] Militello L.G., Hutton R.J.B. (1998). Applied cognitive task analysis (ACTA): a practitioner's toolkit for understanding cognitive task demands. Ergonomics.

[22] Miller J.E., Patterson E.S., Woods D.D. (2001). Critiquing as a cognitive task analysis (CTA) methodology. Proceedings of the Human Factors and Ergonomics Society Annual Meeting.

[23] Yates K.A., Feldon D.F. (2011). Advancing the practice of cognitive task analysis: a call for taxonomic research. Theor. Issues Ergon. Sci..

[24] Papautsky E.L., Dominguez C., Strouse R., Moon B. (2015). Integration of cognitive task analysis and design thinking for autonomous helicopter displays. J. Cognit. Eng. Decis. Mak..

[25] Saitwal H., Feng X., Walji M., Patel V., Zhang J. (2010). Assessing performance of an electronic health record (EHR) using cognitive task analysis. Int. J. Med. Inform..

[26] Clark R.E., Pugh C.M., Yates K.A., Inaba K., Green D.J., Sullivan M.E. (2012). The use of cognitive task analysis to improve instructional descriptions of procedures. J. Surg. Res..

[27] Tofel-Grehl C., Feldon D.F. (2013). Cognitive task analysis–based training: a meta-analysis of studies. J. Cognit. Eng. Decis. Mak..

[28] Hegde S., Gromski M.A., Halic T., Turkseven M., Xia Z., Çetinsaya B., Sawhney M.S., Jones D.B., De S., Jackson C.D. (2020). Endoscopic submucosal dissection: a cognitive task analysis framework toward training design. Surg. Endosc..

[29] Chan C.V., Kaufman D.R. (2011). A framework for characterizing ehealth literacy demands and barriers. J. Med. Internet Res..

[30] Liang S.-F.M., Rau C.-.L., Tsai P.-.F., Chen W.-.S. (2014). Validation of a task demand measure for predicting mental workloads of physical therapists. Int. J. Ind. Ergon..

[31] McCracken J.H., Aldrich T.B. (1984). Analyses of Selected LHX Mission Functions: Implications for Operator Workload and System Automation Goals.

[32] Sutcliffe A. (2003). Scenario-based requirements engineering. Proceedings of the 11th IEEE International Requirements Engineering Conference, 2003.

[33] Salvendy G. (2012). Handbook of Human Factors and Ergonomics, Fourth.

[34] Felipe S.K., Adams A.E., Rogers W.A., Fisk A.D. (2010). Training novices on hierarchical task analysis. Proceedings of the Human Factors and Ergonomics Society Annual Meeting.

[35] Al-Hakim L., Wang M., Xiao J., Gyomber D., Sengupta S. (2019). Hierarchical task analysis for identification of interrelationships between ergonomic, external disruption, and internal disruption in complex laparoscopic procedures. Surg. Endosc..

[36] Yang X., Hyup Kim J., Nazareth R. (2019). Hierarchical task analysis for driving under divided attention. Proceedings of the Human Factors and Ergonomics Society Annual Meeting.

[37] Bloom B.S. (1956). Taxonomy of Educational Objectives: The Classification of Educational Goals.

[38] Harrow A.J. (1972). A Taxonomy of the Psychomotor domain: a Guide For Developing Behavioral Objectives. https://books.google.com/books?id=at87AAAAIAAJ.

[39] Verenna A.-M.A., Noble K.A., Pearson H.E., Miller S.M. (2018). Role of comprehension on performance at higher levels of Bloom's taxonomy: findings from assessments of healthcare professional students. Anat. Sci. Educ..

[40] Crompton H., Burke D., Lin Y.-.C. (2019). Mobile learning and student cognition: a systematic review of PK-12 research using Bloom's taxonomy. Br. J. Educ. Technol..

[41] Anwar F., Sulaiman S., Dominic P.D.D. (2013). Cognitive task analysis: a contextual inquiry study on basic computer and information literacy skills among physicians. Proceedings of the ISICO 2013.

[42] Bertrand A., Mihajlović V., Grundlehner B., Van Hoof C., Moonen M. (2013). Motion artifact reduction in EEG recordings using multi-channel contact impedance measurements. Proceedings of the IEEE Biomedical Circuits and Systems Conference (BioCAS).

[43] Chadwick N.A., McMeekin D.A., Tan T. (2011). Classifying eye and head movement artifacts in EEG signals. Proceedings of the 5th IEEE International Conference on Digital Ecosystems and Technologies (IEEE DEST 2011).

[44] Daly I., Billinger M., Scherer R., Müller-Putz G. (2013). On the automated removal of artifacts related to head movement from the EEG. IEEE Trans. Neural Syst. Rehabil. Eng..

[45] Butkevičiūtė E., Bikulčienė L., Sidekerskienė T., Blažauskas T., Maskeliūnas R., Damaševičius R., Wei W. (2019). Removal of movement artefact for mobile EEG analysis in sports exercises. IEEE Access.

[46] Casson A.J. (2014). Performance of wrist based electrocardiography with conventional ECG analysis algorithms. Proceedings of the 8th Conference of the European Study Group on Cardiovascular Oscillations (ESGCO).

[47] Djamasbi S., Siegel M., Tullis T., Dai R. (2010). Efficiency, trust, and visual appeal: usability testing through eye tracking. Proceedings of the 43rd Hawaii International Conference on System Sciences.

[48] Ikuma L.H., Harvey C., Taylor C.F., Handal C. (2014). A guide for assessing control room operator performance using speed and accuracy, perceived workload, situation awareness, and eye tracking. J. Loss Prev. Process Ind..

[49] Manson S.M., Kne L., Dyke K.R., Shannon J., Eria S. (2012). Using Eye-tracking and Mouse Metrics to Test Usability of Web Mapping Navigation. Cartogr. Geogr. Inf. Sci..

[50] Rubio S., Díaz E., Martín J., Puente J.M. (2004). Evaluation of subjective mental workload: a comparison of SWAT, NASA-TLX, and workload profile methods. Appl. Psychol..

[51] Hart S.G., Staveland L.E., Hancock P.A., Meshkati N. (1988).

[52] AlMousa M., Al-Khalifa H.S., AlSobayel H. (2017). Requirements elicitation and prototyping of a fully immersive virtual reality gaming system for upper limb stroke rehabilitation in Saudi Arabia. Hindawi Mob. Inf. Syst..

[53] Sadiq M., Jain S.K. (2014). Stakeholder identification method in goal oriented requirements elicitation process. Proceedings of the IEEE 5th International Workshop on Requirements Prioritization and Communication (RePriCo).

[54] Singh V., Sankhwar S., Pandey D. (2014). A framework for requirement elicitation. Glob. J. Multidiscip. Stud..

[55] Yousuf M., M.Asger M.A. (2015). Comparison of various requirements elicitation techniques. Int. J. Comput. Appl..

[56] Department of Defense, MIL-STD-1472F, 1998. http://everyspec.com/MIL-STD/MIL-STD-1400-1499/MIL-STD-1472F_208/.

[57] Williams D. (2016). Human Factors Design Guidance for Driver-Vehicle Interfaces.

[58] American National Standard (2006). Human Engineering Design For Marine Systems.

[59] Ogata K., Seya Y., Watanabe K., Ifukube T. (2012). Effects of visual cues on the complicated search task. Proceedings of the 7th Nordic Conference on Human-Computer Interaction Making Sense Through Design - NordiCHI ’12.

[60] Wu X., Li Z. (2018). A review of alarm system design for advanced control rooms of nuclear power plants. Int. J. Hum. Comput. Interact..

[61] Chun J., Han S.H., Park G., Seo J., lee I., Choi S. (2012). Evaluation of vibrotactile feedback for forward collision warning on the steering wheel and seatbelt. Int. J. Ind. Ergon..

[62] Meng F., Gray R., Ho C., Ahtamad M., Spence C. (2015). Dynamic vibrotactile signals for forward collision avoidance warning systems. Hum. Fact. J. Hum. Fact. Ergon. Soc..

[63] Pan Y., Renganayagalu S.K., Komandur S. (2013). Tactile cues for ship bridge operations. Proceedings of the ECMS 2013 Edited by: Webjorn Rekdalsbakken, Robin T. Bye, Houxiang Zhang.

[64] Association for the Advancement of Medical Instrumentation (2010). American National Standards Institute, Human factors engineering: Design of Medical Devices.

[65] J. Yim, R. Myungy, B. Lee, The mobile phone's optimal vibration frequency in mobile environments, in: Usability and Internationalization. HCI and Culture, n.d.: pp. 646–652. https://link-springer-com.proxy-um.researchport.umd.edu/content/pdf/10.1007%2F978-3-540-73287-7.pdf.

[66] Baek Y., Myung R., Yim J. (2006). Have you ever missed a call while moving? The optimal vibration frequency for perception in mobile environments. Proceedings of the 6th WSEAS International Conference on Applied Informatics and Communications.

[67] Hancock P.A., Mercado J.E., Merlo J., Van Erp J.B.F. (2013). Improving target detection in visual search through the augmenting multi-sensory cues. Ergonomics.

[68] R.E. Llaneras, J.P. Singer, Inventory of in-vehicle technology human factors design characteristics: (729332011-001), (2002) 123. https://doi.org/ 10.1037/e729332011-001.

[69] Wilcox S.B. (2011). A human factors perspective : auditory alarm signals. Biomed. Instrum. Technol..

[70] Ho C., Spence C. (2005). Assessing the effectiveness of various auditory cues in capturing a driver's visual attention. J. Exp. Psychol. Appl..

[71] Nees M.A., Helbein B., Porter A. (2016). Speech auditory alerts promote memory for alerted events in a video-simulated self-driving car ride. Hum. Fact. J. Hum. Fact. Ergon. Soc..

[72] Arrabito G.R. (2009). Effects of talker sex and voice style of verbal cockpit warnings on performance. Hum. Fact..

[73] Klingner J., Kumar R., Hanrahan P. (2008). Measuring the task-evoked pupillary response with a remote eye tracker. Proceedings of the 2008 Symposium on Eye Tracking Research & Applications - ETRA ’08.

[74] Mathôt S., Fabius J., Van Heusden E., Van der Stigchel S. (2018). Safe and sensible preprocessing and baseline correction of pupil-size data. Behav. Res..

[75] Xiong H., Pandey G., Steinbach M., Kumar V. (2005). Enhancing Data Analysis With Noise Removal. https://apps.dtic.mil/dtic/tr/fulltext/u2/a439494.pdf.

[76] Hershman R., Henik A., Cohen N. (2018). A novel blink detection method based on pupillometry noise. Behav. Res..

[77] Watson A.B., Yellott J.I. (2012). A unified formula for light-adapted pupil size. J. Vis..

[78] Bretz F., Hothorn T., Westfall P. (2016). Multiple Comparisons Using R.

